# Jacobsen syndrome

**DOI:** 10.1186/1750-1172-4-9

**Published:** 2009-03-07

**Authors:** Teresa Mattina, Concetta Simona Perrotta, Paul Grossfeld

**Affiliations:** 1 Genetica Medica, Department of Pediatrics, University of Catania, Catania, Italy; 2 Division of Pediatric Cardiology, Department of Pediatrics, University of California, San Diego, California, USA

## Abstract

Jacobsen syndrome is a MCA/MR contiguous gene syndrome caused by partial deletion of the long arm of chromosome 11. To date, over 200 cases have been reported. The prevalence has been estimated at 1/100,000 births, with a female/male ratio 2:1. The most common clinical features include pre- and postnatal physical growth retardation, psychomotor retardation, and characteristic facial dysmorphism (skull deformities, hypertelorism, ptosis, coloboma, downslanting palpebral fissures, epicanthal folds, broad nasal bridge, short nose, v-shaped mouth, small ears, low set posteriorly rotated ears). Abnormal platelet function, thrombocytopenia or pancytopenia are usually present at birth. Patients commonly have malformations of the heart, kidney, gastrointestinal tract, genitalia, central nervous system and skeleton. Ocular, hearing, immunological and hormonal problems may be also present. The deletion size ranges from ~7 to 20 Mb, with the proximal breakpoint within or telomeric to subband 11q23.3 and the deletion extending usually to the telomere. The deletion is *de novo *in 85% of reported cases, and in 15% of cases it results from an unbalanced segregation of a familial balanced translocation or from other chromosome rearrangements. In a minority of cases the breakpoint is at the *FRA11B *fragile site. Diagnosis is based on clinical findings (intellectual deficit, facial dysmorphic features and thrombocytopenia) and confirmed by cytogenetics analysis. Differential diagnoses include Turner and Noonan syndromes, and acquired thrombocytopenia due to sepsis. Prenatal diagnosis of 11q deletion is possible by amniocentesis or chorionic villus sampling and cytogenetic analysis. Management is multi-disciplinary and requires evaluation by general pediatrician, pediatric cardiologist, neurologist, ophthalmologist. Auditory tests, blood tests, endocrine and immunological assessment and follow-up should be offered to all patients. Cardiac malformations can be very severe and require heart surgery in the neonatal period. Newborns with Jacobsen syndrome may have difficulties in feeding and tube feeding may be necessary. Special attention should be devoted due to hematological problems. About 20% of children die during the first two years of life, most commonly related to complications from congenital heart disease, and less commonly from bleeding. For patients who survive the neonatal period and infancy, the life expectancy remains unknown.

## Disease name and synonyms

### Jacobsen Syndrome; JBS

Monosomy 11qter

Partial deletion 11q

Distal deletion 11q

Distal monosomy 11q

Telomeric deletion 11q

11q-syndrome

Del 11q ter

Del 11q23.3

## Definition

Jacobsen syndrome (JS) is a contiguous gene syndrome caused by partial deletion of the long arm of chromosome 11. The condition was first described by Jacobsen in 1973 in a family with multiple members that inherited an unbalanced 11;21 translocation derived from a balanced translocation carrier parent [1]. The deletion size ranges from 7 to 20 Mb [2,3]. The breakpoints occur within or distal to subband 11q23.3 and the deletion usually extends to the telomere. Larger terminal deletions extending proximal to 11q23.3 probably result in embryonic lethality, and in some old reports of such deletions the breakpoint might have been misinterpreted due to technical limits of banding resolution [4]. A large terminal deletion with break occurring at band 11q21 has been reported, in the mosaic form, in a patient with a severe complex phenotype with holoprosencephaly and cyclopia [5]. A partial JS phenotype is observed in three patients from a kindred with a very small pure terminal deletions [6]. Partial phenotype is also observed in interstitial deletions rarely occurring within the JS region [7,8].

More commonly, interstitial deletions occur in 11q centromeric to the JS region, giving a distinct clinical phenotype.

The most common clinical features of JS include: pre- and postnatal physical growth retardation, psychomotor retardation, characteristic facial dysmorphism, thrombocytopenia or pancytopenia. A subset of patients have malformations of the heart, kidney, gastrointestinal tract, genitalia, central nervous system and/or skeleton. Ocular, hearing, immunological and hormonal problems may be also present [2,3].

Although patients with the largest deletions usually show more severe clinical manifestations and cognitive impairment, some of the phenotype demonstrate incomplete penetrance and are highly variable between patients.

## Epidemiology

More than 200 cases of JS have been so far reported in the literature [3,9]. The estimated occurrence of JS is about 1/100,000 births [2,3,9]. The female/male ratio is 2:1.

## Clinical description

Data on clinical findings are based on a thorough review of the literature concerning over 200 published cases and personal experience with 62 children with pure 11q terminal deletions (some of whom are also included in above mentioned literature reports) that we have assessed directly. Patients with JS are born at term in more than 60% cases, premature birth occurs in about 30% of cases, while delivery is post term in less than 10% of cases. Complications at delivery occur in 46% of cases: abnormal fetal presentation, premature rupture of the membranes, cephalopelvic disproportion. Delivery is spontaneous in about 65% of cases, whereas Cesarean section is performed in 35% of cases. Birth weight is normal (between the 10^th ^and the 90^th ^percentile) in 60% of babies, below the 10^th ^percentile in 37%, while 3% of children have a birth weight above the 90^th ^percentile. Mean maternal age is 27 years, mean paternal age is 30 years. During the neonatal period, most JS children have prolonged hospitalizations, due most commonly to a combination of feeding difficulties, cardiac problems, and/or bleeding problems. About 20% of children die during the first two years of life, most commonly related to complications from congenital heart disease, and less commonly from bleeding. For patients who survive the neonatal period and infancy, the life expectancy is unknown, Dr. Jacobsen's index case is 45 years old and is living in a group home.

There is a wide spectrum of severity of the clinical phenotype. Half of the patients are diagnosed by age one year of life, usually those with the more obvious clinical features of the disorder, while children with milder features may be diagnosed at an older age.

### Physical growth delay

Height is below the 10^th ^percentile in 75% of cases and in the normal range in 25%. Weight is below the 10^th ^percentile in 58%, normal in 34% and above the 90^th ^percentile in 8% of cases. Head circumference is below the 10^th ^percentile in 26%, normal in 53% and above the 90^th ^percentile in 21% of cases.

### Psychomotor developmental delay

Mental development is normal or borderline in less than 3% of cases, mild to severe mental retardation is observed in 97% of cases. The degree of neurocognitive deficiency is strongly associated with the size of the deletion [3,10]. Children manifest behavioral problems, most frequently attention deficit/hyperactivity disorder, while more severe psychiatric disorders such as schizophrenia, or bipolar affective disorder have been reported rarely [11,12]. Seizures have been reported infrequently.

### Dysmorphic features and minor malformations

**Figure 1:**
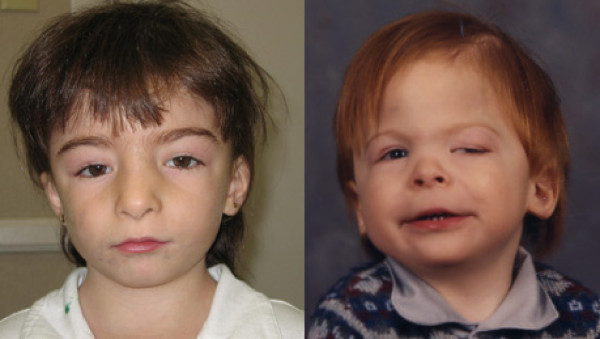
**Facial dysmophism in Jacobsen syndrome patients**.

Typical features are: skull deformities (macrocrania, high prominent forehead, facial asymmetry, trigonocephaly), ocular hypertelorism, downslanting palpebral fissures, strabismus, palpebral ptosis, sparse eyebrows, epicanthal folds, eyelid coloboma, ectropion, iris coloboma, cataract, tortuosity of retinal vessels, flat or prominent nasal bridge, short nose, anteverted nares, prominent columella, broad nasal bridge, small ears, low set posteriorly rotated ears, malformed external ears, hypoplastic lobus, long philtrum, flat philtrum, v-shaped mouth, thin upper lip, retrognathia, and other less frequently observed (Figure 1). Hands show cutaneous syndactyly, flat finger pads with fetal tubercule, thin fingers, large 1^st ^interphalangeal joints, hypoplastic hypotenar regions, abnormal palmar creases, hypoplastic thenar regions. Feet are stubby and flat, with large and long first toe, clynodactylous toes, brachydactyly, syndactyly of the 2^nd ^and 3^rd ^toes, crowded toes. The main features of JS, based on 35 patients that we have assessed, are listed in Table 1. In some reports the presence of trigonocephaly has been described as a common feature. Trigonocephaly is an abnormality of the skull shape characterized by a triangular appearance of the forehead when the head is viewed from above. It is often associated with premature closure of the metopic suture. The presence of trigonocephaly gives the patient a very characteristic facial appearance that raises the possibility of the diagnosis, however in our experience the frequency of trigonocephaly in JS patients seems less high than previously reported (< 30%). The prevalence of trigonocephaly at birth is reported as 67:1,000,000 in the general population; in a series of 25 children selected for the presence of syndromic and non syndromic trigonocephaly, two out of seven syndromic cases (28.5%) were JS patients [13].

**Table 1:** Features in Jacobsen syndrome

	Very frequent (> 40%)	Less frequent (< 40%)
Craniofacial abnormalities	Skull deformities, macrocrania High prominent forehead Facial asymmetry	Trigonocephaly (see Text)

Eyes	Ocular hypertelorism Downslanting palpebral fissures Divergent or convergent strabismus Palpebral ptosis Sparse eyebrows Epicanthal folds	Eyelid coloboma Ectropion Iris coloboma Cataract Tortuosity of retinal vessels

Nose	Flat nasal bridge (early aspect) Prominent nasal bridge (late aspect) Short nose Anteverted nares Prominent columella	Broad nasal bridge

Ears	Small ears Low set ears Posteriorly rotated ears Malformed external ears Hypoplastic lobus	

Mouth	Long philtrum Flat philtrum V-shape mouth Thin upper lip Retrognathia	High arched palate Thick lower lip Dental anomalies

Neck	Short neck	Webbed neck

Hands	Cutaneous syndactyly Flat finger pads with fetal tubercule Thin fingers, large 1^st ^interphalangeal joints Hypoplastic hypotenar regions Abnormal palmar creases	Hypoplastic tenar

Feet	Stubby and flat feet Large and long first toe Clynodactylous toes Brachydactyly Syndactyly of II and III toes	Crowded toes Zygodactyly (toes 2 and 3 very similar)

### Visceral malformations

Congenital heart malformations occur in 56% of cases. Most of these require medications and/or surgical repair. Cardiac malformations were reported in 95% of deceased children [14]. The most frequent heart defects (2/3 of patients that have congenital heart defects) are ventricular septal defects, or left heart obstructive malformations: abnormalities of aortic or mitral valves, coartation of the aorta, Shone's complex, or hypoplastic left heart syndrome. Hypoplastic left heart syndrome, a rare and severe condition that occurs in about 1/5000 infants, is observed in 5% of JS children. The other one third of patients with heart defects have a wide spectrum of most of the common heart defects that occur in the general population of patients with congenital heart disease [3]. Gastrointestinal tract malformations occur in 18% of reported cases, and in 25% the patients we have assessed, including pyloric stenosis, anal abnormalities (atresia or stenosis, or anteriorized anus), and less frequently, duodenal atresia, annular pancreas, or gut malrotation. Functional abnormalities of the gastrointestinal tract also occur frequently, including feeding difficulties in the neonatal period and chronic constipation, requiring medication, beyond the newborn period. Among JS patients who were analyzed with cerebral ultrasound, computed tomography (CT), or magnetic resonance imaging (MRI) scanning or by autopsy, 65% had some kind of structural abnormality of the brain: enlarged ventricles with or without spina bifida, cerebral atrophy, agenesis of corpus callosum, pachygiria [15]. Supratentorial abnormalities of the white matter are interpreted as due to delay of myelinization [14,16,17], or partial loss of astrocytes, rather than a process of demyelinization [9].

Urinary system malformations are observed in 13% of JS children: unilateral kidney dysplasia, double urethers, double kidney district, hydronephrosis, multicystic kidneys. Cryptorchidism is observed in 36% of males in published reports, and in about 60% our series. Inguinal hernias are observed in 15% of children.

In addition to craniosynostosis, 14% of children display some skeletal abnormality including spina bifida occulta, vertebral body anomalies, chest anomalies, abnormal number of ribs, micromelia, hexadactyly. Orthopedics abnormalities are observed in 19% of children including hip dislocation, scoliosis, flat feet, or clubbed feet.

### Hematological, hormonal and immunological aspects

Most patients with the JS are born with either thrombocytopenia or pancytopenia. More recently a definite platelet disorder, the Paris-Trousseau syndrome, has been reported in patients with JS [18,19]. This platelet abnormality is highly penetrant in JS, affecting at least 88.5% of cases, and it has been suggested that the platelet abnormality in JS and the Paris-Trousseau syndrome are the same condition [19,20]: Paris-Trousseau syndrome is characterized by neonatal thrombocytopenia which may resolve over time, and platelet dysfunction (which is usually persistent). In peripheral blood there are two different types of abnormal platelets: giant platelets and platelets with giant alpha granules. Platelets showing giant alpha granules are a minority, but their amount is variable in different patients and in the same patient at different times. Immunocytochemical and ultrastructural studies demonstrated that the giant granules arise by abnormal fusion of smaller organelles [19]. These abnormally fused alpha granules may be due to a failure of the alpha granules to release their contents that are required for coagulation, under normal physiologic conditions. In the bone marrow there is an increased number of small megakaryocytes (micromegakaryocytes), and delayed maturation of megakaryocytes [21]. It has been observed that in JS the platelets have a reduced number of serotonin adenine rich dense bodies, suggesting a storage pool deficit [22].

Growth hormone (IGF-1) and thyroid-stimulating hormone (TSH) deficiency have been reported in patients with JS [9,23].

Although JS infants show recurrent otitis and sinusitis, deficit of cellular or humoral immunity with low IgM and IgA has been reported only occasionally [24,25]. Cutaneous eczema is observed in 22% of JS children, but there is no obvious increased risk for severe allergic reaction to food or medications [3].

### Malignancies

Somatic acquired chromosome deletions of 11q overlapping the JS region have been demonstrated in a variety of malignancies of adulthood, frequently associated with aggressive progression of the disease and poor prognosis. It might be expected, therefore, that constitutional deletions of 11q regions result in an increased risk for developing neoplastic lesions and/or poor prognosis. There is no evidence of increased risk of malignancies in JS patients from the literature, but clinical reports have concerned mainly JS children, and little is known about their outcome in older ages. Better clinical management of JS children leading to longer survivals could reveal an increased risk for the development of malignancies in JS patients in adulthood. Alternatively, it is possible that deletion of 11q terminal region is causally related only with an aggressive progression of cancer rather than with an increased risk of neoplastic transformation and cancer onset [3,26].

## Etiology

In JS the chromosome aberration is usually a *de novo *pure terminal deletion (85% of cases). Less frequently, unbalanced translocations result from segregation of a familial balanced translocation, as in the original report by Jacobsen [1]. Alternatively, partial deletion of chromosome 11q may result from unbalanced translocations occurring *de novo*, or from other chromosomal rearrangements such as ring chromosomes [2,27] or recombination of a parental pericentric inversion. In these cases 11q deletion is complicated by additional imbalances.

Chromosome 11q deletion has also been reported in the mosaic form [5,25,28,29].

**Figure 2:**
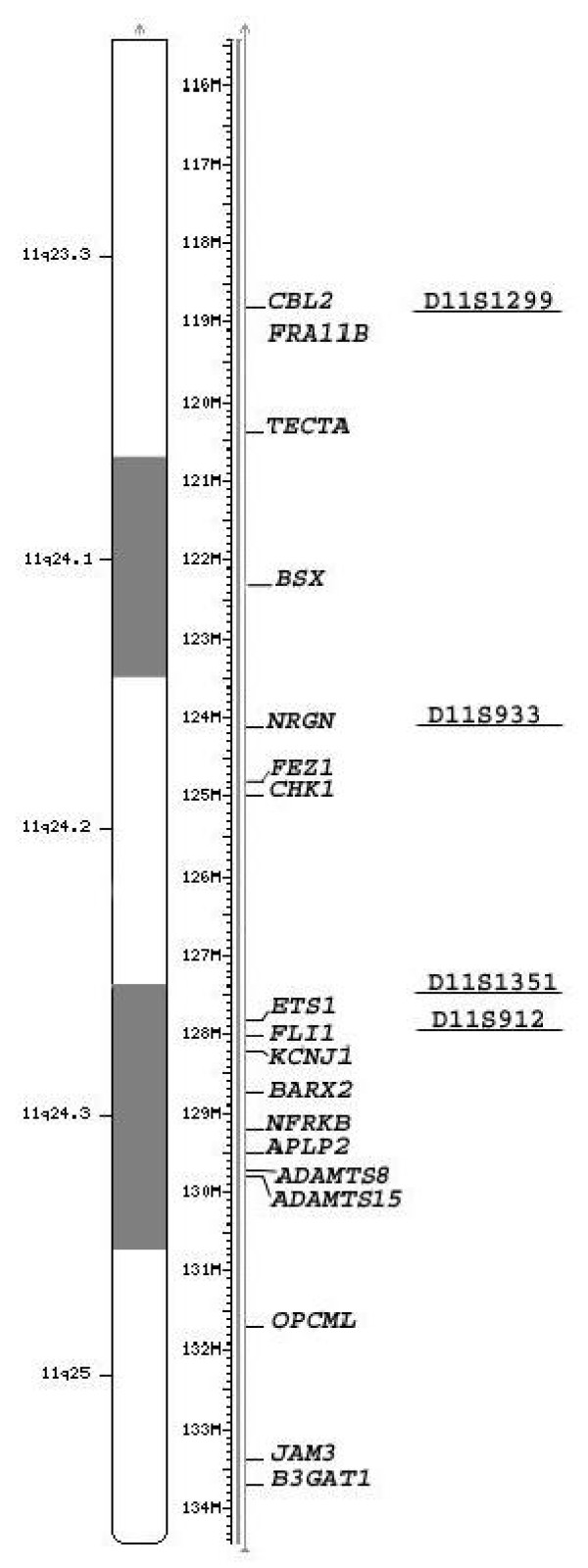
**11q23.3-qter**. From left to right: ideogram, megabase position (Mb), most relevant genes, markers. The position of genes and markers is from UCSC the human march 2006 assembly [32].

In a subset of patients the breakpoints cluster in 11q23.3, close to a rare folate sensitive fragile site *FRA11B *[2,29]. Folate sensitive fragile sites are caused by the extensive expansion of (CGG)n repeats and hypermethylation of adjacent CpG islands. The *FRA11B *site A is a (CGG)n repeat in the 5' untranslated region (UTR) of the *CBL2 *protooncogene [30,31], which maps to between 118, 575, 702 and 118,775,922 bp. [32] (Figure 2). In more than 70% of normal individuals this repeat is present in 11 copies, while in individuals with cytogenetic expression of the *FRA11B *the repeat is expanded to several hundred copies. The CCG repeats can expand to 80–100 copies as a premutation, without cytogenetic expression of the fragile site.

Jones *et al. *reported that in two JS patients the expansion of the CCG repeat at the *FRA11B*, with or without cytogenetic expression of the fragile site, was at the origin of the 11qter deletion. In both cases the deleted chromosome was maternal [30,31]. It is hypothesized that hypermethylation of the expanded (CCG)n triplets on chromosome 11 could delay DNA replication of this fragile site, resulting in a break and/or impaired DNA replication [33].

Only a minority of cases of JS (10%) are related to amplification of the CCG triplet at the *FRA11B *folate sensitive fragile site, and individuals with the *FRA11B *rarely have children with JS. Therefore, the presence of the *FRA11B *fragile site may increase the risk of having JS offspring, but not necessarily result in a child with an 11q deletion. Most 11q deletions occur distally to the *FRA11B*, and it is possible that expansion at other fragile sites may be responsible for distal 11q deletions [33,34]. However, to date no other fragile sites have been identified in the parents of JS patients with deletions whose breakpoints map telomeric to the FRA11B fragile site [3].

Alternatively, smaller deletions are likely to occur by other mechanisms: it has been shown that chromosome recombinations can be mediated by low copy repeats (LCR), palindromic AT-rich repeats (PATRRs), olfactory receptor gene clusters (ORGC) [35-37]. Taken together, there are likely to be multiple mechanisms that lead to the generation of 11q deletions.

In JS patients the origin of the deleted chromosome is more likely to be maternal for breakpoints occurring proximally to D11S924 (band 11q23.3), while there is a paternal bias when the breakpoint is more distal. It is likely that imprinting is involved in the mechanisms of (CCG)n expansion and methylation [2,31].

## Genotype/phenotype correlation

Although the patients with the largest deletions usually show the most severe phenotype, this can vary between patients. For example, as described above, almost all patients have Paris-Trousseau syndrome, but only 56% of patients have a congenital heart defect, independent of the size of their deletion. The minimal region for expressing the JS phenotype spans about 14 Mb [38,39].

Features of JS, resulting in a partial expression of the JS phenotype, have been observed in patients with very small terminal deletions and in cases of interstitial deletions spanning within the JS region [6-8].

A terminal deletion of about 5 Mb, with a breakpoint at 129.5 Mb, was reported in three patients from a kindred, with some facial features of JS, with or without mental retardation, and without cardiac malformation and thrombocytopenia [6].

Some facial features of JS, heart defect and the Paris-Trousseau thrombocytopenia, have been observed in a child with an interstitial deletion of about 10 Mb spanning between 120.0/121.5 and 129.7/130.6, thus almost entirely proximal to that of previous report [7].

Two further patients with interstitial deletions have been reported to show some features of JS, but not the distinct and recognizable facial aspect [8]. The region spanning between 120 and 130.6 Mb is probably the most important for the expression of the phenotype.

Attempts to correlate the clinical findings to the extent of the deletion, complicated by incomplete penetrance for specific phenotypes as described above, has nonetheless provided the following results: Trigonocephaly and skin syndactyly have been localized to a region defined between D11S933 (124 Mb) and D11S912 (128 Mb) [2,32]. Phillips *et al. *(2002) refined the critical region for hypoplastic left heart and trigonocephaly to a region distal to D11S1351 [40], mapping to 127.5–127.7 Mb, according to UCSC database [32]. Grossfeld [3] mapped the Paris-Trousseau platelet disorder, cryptorchidism, pyloric stenosis, and mental retardation to the 6.8 Mb telomeric region spanning from D11S1351 (127.5 Mb) [32]. The region spanning from D11S707 (125.7 Mb) to the telomere is associated with cardiac defects [3], but more than one gene might be involved in cardiac malformation [7,8]. The region distal to D11S1299 (119 Mb) [32] is related to ocular coloboma [3]. Genotype – phenotype correlation has been attempted also in reports concerning patients where 11q terminal deletions are due to the segregation of unbalanced translocations [41-43]. These cases may allow further refinement of the phenotype mapping, however a possible role of the associated duplications must be considered.

## Relevant genes and loci

11q23 qter is a gene rich region: it includes 342 genes, 174 of which have been localized to 11q24.1 qter, the minimal region for expressing the JS, of about 14 Mb [35]. The *FLI-1 *gene maps to chromosome subband 11q24, in the region deleted in JS patients. It is a proto-oncogene belonging to the *ETS *family and interacting with a number of genes involved in vasculogenesis, hematopoiesis and intercellular adhesion. There is *in vivo *and *in vitro *evidence that *FLI1 *plays a fundamental role in megakaryocytes differentiation and that heterozygous loss of the *FLI-1 *gene is associated with dysmegakaryocytopoiesis and the Paris-Tousseau trombocytopenia in JS [19,44]. Furthermore, *in vitro *induced over-expression of the *FLI-1 *gene in patient (*i.e*. patients that are haplo-insufficient for FLI-1) CD34+ cells, restores normal megakaryopoiesis [45], however other genes in distal 11q may also contribute to this disorder. The *BARX 2 *gene maps to chromosome 11q, within the minimal deleted region for JS. It has four exons and encodes a homeobox protein. Its murine homologue *barx2 *is expressed in the development of neural and craniofacial structures [46]. The *BARX 2 *gene, because of its location and its expression pattern, is a possible candidate gene for the development of facial dysmorphism and/or craniosynostosis in JS [46]. However no mutation in the *BARX2 *gene has been detected in nine patients with isolated trigonocephaly [46]. *JAM3 *has been considered a candidate gene for hypoplastic left heart and trigonocephaly. It is a member of the junction adhesion molecules family; the gene maps within the critical region for hypoplastic left heart (HLH) that is a 9 Mb region in the long arm of chromosome 11 distal to D11S1351 (127.5 Mb). Analysis of the *JAM3 *gene in thirty-three patients with isolated HLH, failed to demonstrate any mutation; this would suggest that other genes are mainly involved in this malformation [7]. Tyson *et al. *[8], suggested that a critical region for the conotruncal heart defect may lie within a region spanning between 129.03 and 130.6 Mb, which bears *ADAMTS8*, a gene involved in angiogenesis.

*B3GAT1*, a beta-1–3-glycoronyltransferase gene located in 11q25 at 133.77 Mb, is expressed in the brain. Knock out mice show impaired synaptic plasticity and learning disabilities [47]. This gene could be involved in bipolar disorder observed in some JS patients [12].*BSX*, an evolutionarily highly conserved homeobox gene, expressed during early brain development and mapping in 122.3 Mb has been proposed as a candidate gene for global cognitive development, while *NRGN *(neurogranin), a gene involved in synapse plasticity and long-term potentiation and mapping in 124.1 Mb has been suggested to contribute to the auditory attention deficit [10]. Abnormalities of the white matter appear to map between 124.6 Mb, and 129.03 and hypothetical candidate genes include *FEZ1*, involved in axonal outgrowth, and *RICS*, highly expressed in developing brain [8].

*KCNJ*1, a potassium channel, and *ADAMTS15*, a zinc-dependent proteases expressed in fetal liver and kidney may be related to kidney malformations [8].

*TECTA*, a gene coding for a non collagenous component of the tectorial membrane of the inner ear, may be involved in neurosensorial deafness [48].

A number of tumor related genes map in 11q distal region: *EST1, CHK1, BARX2, OPCML*, *FLI-1*, their role in tumor development and progression in JS is still unknown (Figure 2).

## Diagnosis

In patients with the classical phenotype the diagnosis is suspected on the basis of clinical findings: mental retardation, facial dysmorphic features and thrombocytopenia. The diagnosis must be confirmed by cytogenetic analysis. The clinical diagnosis may be difficult in patients with less characteristic clinical aspects and borderline mental development; in these cases the observation of thrombocytopenia or pancytopenia may suggest the diagnosis. Children with fewer manifestations of JS may be less likely to be diagnosed early, based on clinical phenotype.

## Differential diagnosis

Children with JS share some clinical features (short stature, short, wide, sometimes webbed neck, downslanting palpebral fissures, ptosis, aortic or pulmonary stenosis) with Turner and Noonan syndromes. In Noonan syndrome patients, common features with JS patient are also thrombocytopenia and bleeding tendency. Occasionally, JS children have had a clinical diagnosis of Kabuki syndrome (mental retardation, unusual palpebral fissures, short stature, fingerpads). Neonatal bleeding and thrombocytopenia may be misdiagnosed as acquired thrombocytopenia due to sepsis.

## Genetic counseling

In families with a *de novo *chromosome rearrangement the recurrence risk is generally considered negligible. However in *de novo *terminal deletions 11q, recurrence has been documented in at least four children from two unrelated families [3,49]. Therefore prenatal diagnosis is warranted as the possibility of gonadal mosaicism or any other predisposing condition in one parent cannot be excluded. In families with the fragile site *FRA11B*, the recurrence risk is non quantifiable but probably slightly increased, and prenatal diagnosis could be considered. When a parent is affected with JS [17,18], the recurrence risk is 50%. There is a high recurrence risk when a parent has a balanced translocation, or deletion 11q in the mosaic form.

## Antenatal diagnosis

Only a few prenatal cases of 11q terminal deletion have been reported [50-52]. Prenatal diagnosis of 11q deletion is possible by amniocentesis or chorionic villus sampling and cytogenetic analysis with standard G-banding and, if necessary, telomeric FISH. The test is indicated when there is a known risk for 11q deletion (familial balanced translocation, mosaicism or *FRA11B *in a parent). The cytogenetic test may also be performed when there is an abnormal prenatal serum screening test for Down syndrome or abnormal prenatal ultrasound that may suggest the occurrence of a chromosome unbalance. In some cases of deletion 11q, oligohydramnios, nuchal thickening, heart malformations and kidney duplication have been observed prenatally.

## Management and treatment

After the diagnosis of JS a complete evaluation should be performed including:

• clinical assessment by a general pediatrician

• baseline evaluation by a pediatric cardiologist, including EKG and echocardiogram

• baseline evaluation by a neurologist, including baseline, and potentially serial brain imaging studies

• abdominal ultrasound scan to exclude pyloric stenosis, and kidney and urinary tract malformations.

• ophthalmologic examination, including dilation of the pupils

• auditory tests

• blood tests: blood cells counts, platelets counts, platelets function studies, bleeding time

• endocrine: IGF1 and TSH

• immunological assessment: IgM, IgA, IgG.

*In the neonatal period *children with JS frequently require special assistance and treatment. Cardiac malformations can be very severe and require heart surgery in the neonatal period. JS children may have difficulties in feeding and tube feeding may be necessary. Special attention should be devoted due to hematological problems. Due to thrombocytopenia and abnormal platelet function, bleeding is most likely to occur in infancy. Platelet count can eventually reach low normal values, but functional abnormalities usually persist. In some patients pancytopenia is observed. Prophylactic platelets or whole blood transfusion may be necessary before, as well as during or after surgery. Bone marrow biopsy is usually not necessary. Early craniotomy is indicated in children with craniosynostosis.

### At the follow up

pediatric evaluation, serial blood tests including platelets counts, cardiac evaluation (for the development of progressive lesions such as aortic or mitral valve stenosis), ophthalmologic and auditory tests should be performed, and can be individualized to each patient's needs. During the first years of life children have an increased risk of recurrent infections. Thyroid hormone deficiency should be treated accordingly. Most JS patients have short stature, half of whom have growth hormone (IGF-1) deficiency. Treatment with growth hormone replacement therapy is controversial, since these patients may have a genetic predisposition to certain malignancies that could be exacerbated by a tumor promoter. Eye surgery may be necessary to correct strabismus, as untreated strabismus may result in amblyopia. It is critical that surgery be performed in the first year of life to prevent this complication.

Severe ptosis must be corrected to allow walking; however abnormal external eye muscles may cause relapse of ptosis or strabismus after surgery. Treatment of orthopedic problems should be individualized. Early intervention with occupation, speech, physical and behavioral therapists is critical to address cognitive and behavioral problems. Recently, music therapy has been shown to be beneficial to some of these patients (manuscript in preparation). JS children can be vaccinated according to the standard schedule [3].

### Surgery

As described above, children with JS frequently require surgical treatment interventions. Thrombocytopenia and other hematological problems must be taken into account preoperatively. Prophylactic transfusion with platelets can be lifesaving. Due to abnormalities of the pharynx maintaining the airway and intubation might be difficult [53].

## Prognosis

A proportion of children with JS die in the neonatal period, usually due to severe heart malformations and bleeding. Many patients that survive the neonatal period will require long-term care including surgical and medical interventions. Life expectancy is unknown. The oldest known living patient with JS is 45 years old. There is apparently no known increased risk of malignancies.

## List of abbreviations

JS: Jacobsen Syndrome; HLH: Hypoplastic Left Heart; LCR: Low Copy Repeats; PATRRs: Palindromic AT-Rich Repeats; ORGC: Olfactory Receptor Gene Cluster.

## Consent

Written consent for publication of photographs was obtained from the patients or legal guardians where required.

## Competing interests

The authors declare that they have no competing interests.

## Authors' contributions

TM conceived of the study, participated in its design and coordination, and helped to draft the manuscript. CSP participated in the design of the study and in the draft of the manuscript. PG has been involved in revising the manuscript. All authors read and approved the final manuscript.

## References

[B1] Jacobsen P, Hauge M, Henningsen K, Hobolth N, Mikkelsen M, Philip J (1973). An (11;21) translocation in four generations with chromosome 11 abnormalities in the offspring. A clinical, cytogenetical, and gene marker study. Hum Hered.

[B2] Penny LA, Dell'Aquila M, Jones MC, Bergoffen J, Cunnif C, Fryns JP, Grace E, Graham JM, Kouseff B, Mattina T, Syme J, Voullaire L, Zelante L, Zenger-Hain J, Jones OW, Evans GA (1995). Clinical and molecular characterization of patients with distal 11q deletion. Am J Hum Genet.

[B3] Grossfeld PD, Mattina T, Lai Z, Favier R, Jones KL, Cotter F, Jones C (2004). The 11q terminal deletion disorder: a prospective study of 110 cases. Am J Med Genet.

[B4] Schinzel A, Auf de Maur P, Moser H (1977). Partial deletion of long arm of chromosome 11 del 11q23: Jacobsen syndrome. J Med Genet.

[B5] Helmuth RA, Weaver DD, Wills ER (1989). Holoprosencephaly, ear abnormalities, congenital heart defect and microphallus in a patient with 11q- mosaicism. Am J Med Genetic.

[B6] Bernaciak J, Szczałuba K, Derwiñska K, Wiśniowiecka-Kowalnik B, Bocian E, Sasiadek MM, Makowska I, Stankiewicz P, Smigiel R (2008). Clinical and molecular-cytogenetic evaluation of a family with partial Jacobsen syndrome without thrombocytopenia caused by an approximately 5 Mb deletion del(11)(q24.3). Am J Med Genet A.

[B7] Wenger SL, Grossfeld PD, Siu BL, Coad JE, Keller FG, Hummel M (2006). Molecular characterization of an 11q interstitial deletion in a patient with the clinical features of Jacobsen syndrome. Am J Med Genet.

[B8] Tyson C, Qiao Y, Harvard C, Liu X, Bernier FP, McGillivray B, Farrell SA, Arbour L, Chudley AE, Clarke L, Gibson W, Dyack S, McLeod R, Costa T, Vanallen MI, Yong SL, Graham GE, Macleod P, Patel MS, Hurlburt J, Holden JJ, Lewis SM, Rajcan-Separovic E (2008). Submicroscopic deletions of 11q24–25 in individuals without Jacobsen syndrome: re-examination of the critical region by high-resolution array-CGH. Mol Cytogenet.

[B9] Pivnick EK, Velagaleti GV, Wilroy RS, Smith ME, Rose ME, Tipton RE, Tharapel AT (1996). Jacobsen Syndrome: Report of a patient with severe eye anomalies, growth hormone seficiency, and hypotiroidism associated with deletion 11 (q23q25) and review of 52 cases. J Med Genet.

[B10] Coldren CD, Lai Z, Shragg P, Rossi E, Glidewell SC, Zuffardi O, Mattina T, Ivy DD, Curfs LM, Mattson SN, Riley EP, Treier M, Grossfeld P (2008). Chromosomal microarray mapping suggests a role for BSX and Neurogranin in neurocognitive and behavioural defects in the 11q terminal deletion disorder (Jacobsen syndrome). Neurogenetics.

[B11] Neavel CB, Soukup S (1994). Deletion of (11)(q24.2) in a mother and daughter with similar phenotypes. Am J Med Genet.

[B12] Böhm D, Hoffmann K, Laccone F, Wilken B, Dechent P, Frahm J, Bartels I, Bohlander SK (2006). Association of Jacobsen Syndrome and bipolar affective disorder in a patient with a de novo 11q terminal deletion. Am J Med Genet.

[B13] Azimi C, Kennedy SJ, Chitayat D, Chakraborty P, Clarke JT, Forrest C, Teebi AS (2003). Clinical and genetic aspects of trigonocephaly: a study of 25 cases. Am J Med Genet.

[B14] Leegte B, Kerstjens-Frederikse WS, Deelstra K, Begeer JH, van Essen AJ (1999). 11q- syndrome: three cases and a review of the literature. Genet Couns.

[B15] Lin JH, Hou JW, Teng RJ, Tien HF, Lin KH (1998). Jacobsen distal 11q deletion syndrome with myelodysplatic change of haemopoietic cells. Am J Med Genet.

[B16] Wardinsky TD, Weinberger E, Pagon RA, Clarren SK, Thuline HC (1990). Partial deletion of the long arm of chromosome 11 [del(11)(q23.3----qter)] with abnormal white matter. Am J Med Genet.

[B17] Ono J, Hasegawa T, Sugama S, Sagehashi N, Hase Y, Oku K, Endo Y, Ohdo S, Ishikiriyama S, Tsukamoto H, Okada S (1996). Partial deletion of the long arm of chromosome 11: ten Japanese children. Clin Genet.

[B18] Favier R, Douay L, Esteva B, Portnoi MF, Gaulard P, Lecompte T, Perot C, Adam M, Lecrubier C, Akker J Van den (1993). A novel genetic thrombocytopenia (Paris-Trousseau) associated with platelet inclusions, dysmegakaryopoiesis and chromosome deletion AT 11q23. C R Acad Sci III.

[B19] Favier R, Jondeau K, Boutard P, Grossfeld P, Reinert P, Jones C, Bertoni F, Cramer EM (2003). Paris-Trousseau syndrome: clinical, haematological, molecular data of ten new cases. Thromb Haemost.

[B20] Krishnamurti L, Neglia JP, Nagarajan R, Berry SA, Lohr J, Hirsch B, White JG (2001). Paris-Trousseau syndrome platelets in a child with Jacobsen's syndrome. Am J Med Haematol.

[B21] Gangarossa S, Schiliró G, Mattina T, Scardilli S, Mollica F, Cavallari V (1996). Dysmegakaryopoietic thrombocytopenia in patients with distal chromosome 11q deletion. Blood.

[B22] White JG (2007). Platelet storage pool deficiency in Jacobsen syndrome. Platelets.

[B23] Haghi M, Dewan A, Jones KL, Reitz R, Jones C, Grossfeld P (2004). Endocrine abnormalities in patients with Jacobsen (11q-) syndrome. Am J Med Genet A.

[B24] Sirvent N, Monpoux F, Pedeutour F, Fraye M, Philip P, Ticchioni M, Turc-Carel C, Mariani R (1998). Jacobsen's syndrome, thrombopenia and humoral immunodeficiency. Arch Pediatr.

[B25] von Bubnoff D, Kreiss-Nachtsheim M, Novak N, Engels E, Engels H, Behrend C, Propping P, de la Salle H, Bieber T (2004). Primary immunodeficiency in combination with transverse upper limb defect and anal atresia in a 34-year-old patient with Jacobsen syndrome. Am J Med Genet.

[B26] Koreth J, Bakkenist CJ, McGee JO (1999). Chromosomes, 11q and cancer: a review. J Pathol.

[B27] Palka G, Verrotti A, Peca S, Mosca L, Lombardo G, Verrotti M, Morgese G (1986). Ring chromosome 11. A case report and review of the literature. Ann Genet.

[B28] Kuster W, Gebauer HS, Majewski F, Lenard HG (1985). Report of a deletion 11 (qter->q23.3) and short review of the literature. Eur J Pediatr.

[B29] Voullaire LE, Webb GC, Leversha MA (1987). Chromosome deletion at 11q23 in an abnormal child from a family with inherited fragility at 11q23. Hum Gene.

[B30] Jones C, Penny L, Mattina T, Yu s, Baker E, Voullaire L, Langdon WY, Sutherland GR, Richards RI, Tunnacliffe A (1995). Association of a chromosome deletion syndrome with a fragile site within the proto-oncogene CBL2. Nature.

[B31] Jones C, Müllenbach R, Grossfeld P, Auer R, Favier R, Chien K, James M, Tunnacliffe A, Cotter F (2000). Co-localisation of CCG repeats and chromosome deletion breakpoints in Jacobsen syndrome: evidence for a common mechanism of chromosome breakage. Hum Mol Genet.

[B32] UCSC. The human march 2006 assembly.

[B33] Michaelis RC, Velagaleti GV, Jones C, Pivnick EK, Phelan MC, Boyd E, Tarleton J, Wilroy RS, Tunnacliffe A, Tharapel AT (1998). Most Jacobsen syndrome deletion breakpoints occur distal to FRA11B. Am J Med Genet.

[B34] Tunnacliffe A, Jones C, Le Paslier D, Todd R, Cherif D, Birdsall M, Devenish L, Yousry C, Cotter FE, James MR (1999). Localization of Jacobsen syndrome breakpoints on a 40-Mb physical map of distal chromosome 11q. Genome Res.

[B35] Edelmann L, Spiteri E, McCain N, Goldberg R, Pandita RK, Duong S, Fox J, Blumenthal D, Lalani SR, Shaffer LG, Morrow BE (1999). A common breakpoint on 11q23 in carriers of the constitutional t(11, 22) translocation. Am J Hum Genet.

[B36] Kurahashi H, Shaikh TH, Zackai EH, Celle L, Driscoll DA, Budarf ML, Emanuel BS (2000). Tightly Clustered 11q23 and 22q11 Breakpoints Permit PCR-Based Detection of the Recurrent Constitutional t(11;22). Am J Hum Genet.

[B37] Giglio S, Broman KW, Matsumoto N, Calvari V, Gimelli G, Neumann T, Ohashi H, Voullaire L, Larizza D, Giorda R, Weber JL, Ledbetter DH, Zuffardi O (2001). Olfactory receptor gene clusters, genomic-inversion polymorphism, and common chromosome rearrangements. Am J Hum Genet.

[B38] Fryns JP, Kleczkowska A, Buttiens M, Marien P, Berghe H van den (1986). Distal 11q monosomy. The typical 11q monosomy syndrome is due to deletion of subband 11q24.1. Clin Genet.

[B39] NCBI map viewer. http://www.ncbi.nlm.nih.gov/projects/mapview/.

[B40] Phillips HM, Renforth GL, Spalluto C, Hearn T, Curtis AR, Craven L, Havarani B, Clement-Jones M, English C, Stumper O, Salmon T, Hutchinson S, Jackson MS, Wilson DI (2002). Narrowing the critical region within 11q24-qter for hypoplastic left heart and identification of a candidate gene, JAM3, expressed during cardiogenesis. Genomics.

[B41] Zahn S, Ehrbrecht A, Bosse K, Kalscheuer V, Propping P, Schwanitz G, Albrecht B, Engels H (2005). Further delineation of the phenotype maps for partial trisomy 16q24 and Jacobsen syndrome by a subtle familial translocation t(11;16)(q24.2;q24.1). Am J Med Genet A.

[B42] Podraza J, Fleenor J, Grossfeld P (2007). An 11q terminal deletion and tetralogy of Fallot. Am J Med Genet A.

[B43] Courtens W, Wauters J, Wojciechowski M, Reyniers E, Scheers S, van Luijk R, Rooms L, Kooy F, Wuyts W (2007). A de novo subtelomeric monosomy 11q (11q24.2-qter) and trisomy 20q (20q13.3-qter) in a girl with findings compatible with Jacobsen syndrome: case report and review. Clin Dysmorphol.

[B44] Hart A, Melet F, Grossfeld P, Chien K, Jones C, Tunnacliffe A, Favier R, Bernstein A (2000). Fli-1 is required for murine vascular and megakaryocytic development and is hemizygously deleted in patients with thrombocytopenia. Immunity.

[B45] Raslova H, Favier R, Albagli O, Vainchenker W (2004). Fli1 haploinsufficiency underlies Paris-Trousseau thrombocytopenia. Med Sci (Paris).

[B46] Krasner A, Wallace L, Thiagalingam A, Jones C, Lengauer C, Minahan L, Ma Y, Kalikin L, Feinberg AP, Jabs EW, Tunnacliffe A, Baylin SB, Ball DW, Nelkin BD (2000). Cloning and chromosomal localization of the human BARX2 homeobox protein gene. Gene.

[B47] Yamamoto S, Oka S, Inoue M, Shimuta M, Manabe T, Takahashi H, Miyamoto M, Asano M, Sakagami J, Sudo K, Iwakura Y, Ono K, Kawasaki T (2002). Mice deficient in nervous system-specific carbohydrate epitope HNK-1 exhibit impaired synaptic plasticity and spatial learning. J Biol Chem.

[B48] Hughes DC, Legan PK, Steel KP, Richardson GP (1998). Mapping of the alpha-tector in gene (TECTA) to mouse chromosome 9 and human chromosome 11: a candidate for human autosomal dominant nonsyndromic deafness. Genomics.

[B49] Afifi HH, Zaki MS, El-Gerzawy AM, Kayed HF (2008). Distal 11q monosomy syndrome: a report of two Egyptian sibs with normal parental karyotypes confirmed confirmed by molecular cytogenetics. Genet Couns.

[B50] Chen CP, Chern SR, Chang TY, Tzen CY, Lee CC, Chen WL, Chen LF, Wang W (2004). Prenatal diagnosis of the distal 11q deletion and review of the literature. Prenat Diagn.

[B51] Valduga M, Cannard VL, Philippe C, Romana S, Miton A, Droulle P, Foliguet B, Lecompte T, Jonveaux P (2007). Prenatal diagnosis of mosaicism for 11q terminal deletion. Eur J Med Genet.

[B52] Boehm D, Laccone F, Burfeind P, Herold S, Schubert C, Zoll B, Männer J, Pauer HU, Bartels I (2006). Prenatal diagnosis of a large de novo terminal deletion of chromosome 11q. Prenat Diagn.

[B53] Blaine Easley R, Sanders D, McElrath-Schwartz J, Martin J, Mark Redmond J (2006). Anesthetic implications of Jacobsen syndrome. Pediatr Anesthesia.

